# Effectiveness of baricitinib versus sarilumab on disease activity in patients with RA: a propensity score matching study

**DOI:** 10.1093/rap/rkaf006

**Published:** 2025-01-10

**Authors:** Toshitaka Yukishima, Yukio Nakamura, Shin-Ichiro Ohmura, Tomonori Kobayakawa

**Affiliations:** Department of Rheumatology, Seirei Hamamatsu General Hospital, Shizuoka, Japan; Department of Orthopedic Surgery, Division of Osteoporosis, Locomotive Syndrome, Joint Disease Center, Aichi Medical University, Aichi, Japan; Department of Rheumatology, Seirei Hamamatsu General Hospital, Shizuoka, Japan; Kobayakawa Rheumatology Orthopedic Clinic, Shizuoka, Japan

**Keywords:** RA, Janus kinase inhibitors, baricitinib, sarilumab, propensity score matching

## Abstract

**Objectives:**

To evaluate the effects of baricitinib, a Janus kinase inhibitor, versus sarilumab, a human monoclonal antibody against the IL-6 receptor, on the disease activity of patients with RA.

**Methods:**

At our hospital and cooperative facilities, we initiated treatment with baricitinib and sarilumab and observed patients with RA longitudinally for 52 weeks. Propensity score matching (age, sex, disease duration, MTX/glucocorticoid usage, RF/ACPA positivity and Disease Activity Score 28 with CRP level) was performed to address potential treatment selection bias, resulting in 46 patients in each group. The following data were collected: Disease Activity Score 28 with CRP, Clinical Disease Activity Index, Simplified Disease Activity Index, Boolean 2.0 and their component indices at weeks 24 and 52. A comparative analysis was conducted between the two groups.

**Results:**

Compared with baricitinib, sarilumab induced a similar improvement in disease activity; however, baricitinib induced a significantly greater improvement in the Clinical Disease Activity Index at 24 weeks than sarilumab. At the component level, baricitinib significantly improved the number of swollen joints at 24 weeks, improving the Clinical Disease Activity Index; however, after 52 weeks, the difference between the two groups was no longer statistically significant.

**Conclusion:**

Compared with sarilumab, baricitinib improved swollen joints and the Clinical Disease Activity Index at 24 weeks; however, by 52 weeks, no significant difference was observed between the two groups, indicating that both treatments are important for long-term use.

Key messages

- Baricitinib showed notable short-term improvement in the Clinical Disease Activity Index at 24 weeks.
- Baricitinib significantly improves swollen joint counts at 24 weeks.
- Both baricitinib and sarilumab show equivalent long-term effectiveness at 52 weeks.

## Introduction

In the management of RA, achieving symptom relief for all patients remains challenging, even with recent advances in treatment strategies. The EULAR guidelines emphasize a treat-to-target approach, which includes early diagnosis, timely initiation and adjustment of DMARDs, progressing to biological (b) DMARDs and targeted synthetic (ts) DMARDs as needed [1]. Despite the availability of advanced therapies targeting various cytokines and signalling pathways, many RA patients continue to experience inadequate symptom control. In response, EULAR has identified ‘difficult-to-treat RA’ (D2T RA) as a distinct subgroup, officially defined in 2020, with specific management guidelines published in 2022 [2, 3]. However, there remains a lack of cohort studies that directly compare the effectiveness of different therapeutic agents for RA, especially in real-world settings.

One such treatment of RA, sarilumab, specifically binds to soluble and membrane-associated IL-6 receptors. Compared with adalimumab in patients with RA who did not respond adequately to MTX, sarilumab demonstrated a significantly greater improvement in disease activity by 24 weeks [4]. A recent study showed that the safety profiles and laboratory findings of patients treated with sarilumab were comparable to those observed in individuals receiving tocilizumab therapy [5].

Baricitinib, a targeted inhibitor of Janus kinase (JAK)1 and JAK2, blocks the activity of various cytokines that rely on JAK signalling, including IL-6 [6]. The RA-BEAM trial, a renowned study comparing the efficacy of baricitinib with adalimumab, revealed that in patients with RA who did not achieve an adequate response to MTX, baricitinib demonstrated a significantly higher achievement rate in the ACR 20, at as early as 12 weeks compared with adalimumab, and maintained the equivalent significant efficacy at 52 weeks [7]. IL-6 inhibitors theoretically exclusively target IL-6, whereas baricitinib selectively inhibits downstream JAK1 and JAK2 in the same cascade, potentially offering a similar signalling pathway. Previous studies compared the disease activity of tocilizumab and baricitinib [8].

Considering the lack of studies directly comparing sarilumab and baricitinib with matched patient backgrounds, this study aimed to utilize the propensity score matching technique to fill this gap in the literature. This methodological approach enhanced the validity of our comparisons, making our findings particularly relevant for clinical decision-making and future research on treatment strategies for RA.

## Methods

### Patients and data collection

We included Japanese patients with RA who received baricitinib or sarilumab at our hospital and two collaborating institutions, all located in Japan, between September 2017 and April 2023. RA was diagnosed based on either the 1987 RA classification criteria of the ACR [9] or the 2010 ACR/EULAR RA classification criteria [10]. Most patients in this study presented with moderate disease activity at baseline, as defined by Disease Activity Score 28 (DAS28), Clinical Disease Activity Index (CDAI) and Simplified Disease Activity Index (SDAI) scores. Additionally, a substantial proportion of patients were classified as csDMARDs-inadequate responders or b/tsDMARDs-inadequate responders, reflecting a patient population commonly seen in real-world clinical practice. The baseline demographic data, such as age, sex, disease duration, previous use of bDMARDs/tsDMARDs, usage of MTX and glucocorticoids (GCs), RF and ACPA positivity, tender joint count (TJC), swollen joint count (SJC), CRP, MMP-3, Patient Global Assessment (PGA), Evaluator Global Assessment (EGA), DAS28 with CRP (DAS28-CRP), CDAI, SDAI, Boolean remission criteria 2.0 and Health Assessment Questionnaire Disability Index (HAQ-DI), were collected.

### Statistical analysis

Because of the retrospective and observational nature of this study, we conducted it using the available number of cases and did not perform any sample size calculations. To address potential treatment selection bias, we employed propensity score matching (PSM) to balance the baseline characteristics between the patient groups treated with baricitinib and sarilumab. A propensity score was estimated using a multivariate logistic regression model to predict treatment with baricitinib versus sarilumab using the following variables: age, sex, disease duration, MTX/GC usage, RF/ACPA positivity and DAS28-CRP level. The pairing was achieved with a calliper tolerance of 20% of the S.D. of the propensity score, and random selection was performed among patients with the same propensity score. Ultimately, 46 pairs of patients were matched.

Patient background characteristics between the two groups were compared using the *t*-test and chi-square test before and after propensity score matching, respectively. Disease activity and clinical and laboratory activities were compared between the two treatment groups using Wilcoxon’s rank sum test. Differences between baseline and 24/52 weeks were tested using Wilcoxon’s signed-rank test. Kaplan–Meier survival analysis was conducted to compare drug retention rates over the 52-week period between the two groups. Differences between groups were assessed using the log-rank test.

The last observation-carried forward method (LOCF) was used for each analysis. At baseline, there were no missing data for the primary outcome measures. In the sarilumab group, one patient had a missing disease activity score at the 52-week time point, which was handled using LOCF. The 95% confidence intervals for all estimated effect sizes and measures of association are presented. Two-sided *P*-values were calculated for all hypothesis tests, with *P* < 0.05 being considered significant. Statistical analyses were performed using EZR (Saitama Medical Center, Jichi Medical University, Saitama, Japan), a graphical user interface for the R software (The R Foundation for Statistical Computing, Vienna, Austria) [11].

### Ethical approval

This study was approved by the Ethics Committee of Seirei Hamamatsu General Hospital (4514) and complied with the principles of the Declaration of Helsinki. The committee waived the requirement for patient informed consent by posting the opt-out information on the hospitals’ home page. Patient anonymity was maintained during data collection, and personal information security was strictly controlled.

## Results

The baseline characteristics of all patients included in this study, both before and after propensity score matching, are presented in Table 1. Before matching, the baricitinib group was older (mean±S.D., 69.2 ± 13.0 vs. 65.2 ± 13.9 years, *P* = 0.049), and the sarilumab group had a longer duration of illness (49.5 ± 81.0 vs. 99.1 ± 110.1 months, *P* = 0.001). At baseline, most patients presented with moderate disease activity according to DAS28, CDAI and SDAI scores, and a substantial proportion of patients were classified as csDMARDs-inadequate responders or b/tsDMARDs-inadequate responders, reflecting the real-world applicability of our study. After matching, differences in patient backgrounds were adjusted, allowing the extraction of two groups of patients that were approximately the same; however, it is worth noting that, although there was no significant difference in the use of MTX, it was more commonly co-administered with sarilumab.

**Table 1.** Baseline characteristics before and after propensity score matching

Characteristics	Before matching			After matching		
	Baricitinib (*n* = 67)	Sarilumab (*n* = 140)	*P*-value	Baricitinib (*n* = 46)	Sarilumab (*n* = 46)	*P*-value
Age (years)	69.2 ± 13.0	65.2 ± 13.9	0.049	68.5 ± 12.8	65.4 ± 16.5	0.312
Female	80.6%	76.4%	0.593	76.1%	76.1%	>0.999
Disease duration (months, median [IQR])	20.00 [5.00, 57.50]	50.50 [8.00, 123.00]	0.011	28.0 [11.0, 75.0]	60.5 [13.5, 139.5]	0.098
Use of b/tsDMARDs	65.7%	67.9%	0.754	76.1%	73.9%	>0.999
Median number of previous b/tsDMARDs	1 (range: 0–5)	1 (range: 0–5)		2 (range: 0–5)	2 (range: 0–5)	
Median number of previous TNF inhibitors	1 (range: 1–3)	2 (range: 1–3)		1 (range: 1–3)	2 (range: 1–3)	
Use of MTX	46.3%	38.6%	0.296	43.5%	65.2%	0.059
MTX dose (mg/week)	10.1 ± 3.3	9.1 ± 3.4	0.179	10.2 ± 3.0	9.2 ± 3.5	0.324
Use of GCs	34.3%	25.7%	0.249	28.3%	28.3%	>0.999
RF positivity	73.1%	65.7%	0.336	73.9%	76.1%	0.303
ACPA positivity	84.0%	70.3%	0.063	84.8%	95.7%	0.158
TJC (0–28 scale)	4.4 ± 6.4	5.1 ± 6.7	0.462	5.1 ± 7.3	5.4 ± 6.5	0.833
SJC (0–28 scale)	4.8 ± 5.9	4.5 ± 4.7	0.669	5.7 ± 6.7	5.4 ± 4.6	0.746
CRP (mg/dl)	1.7 ± 2.4	1.76 ± 9.0	0.959	1.8 ± 2.7	1.9 ± 2.2	0.847
MMP-3 (ng/ml)	179.3 ± 208.5	155.0 ± 167.2	0.375	165.7 ± 155.6	197.9 ± 152.5	0.351
PGA (0–100 mm scale)	38.1 ± 26.1	39.2 ± 29.4	0.801	38.0 ± 27.6	46.6 ± 30.5	0.113
EGA (0–100 mm scale)	37.0 ± 23.6	39.8 ± 24.8	0.526	39.1 ± 25.9	47.4 ± 22.0	0.174
DAS28-CRP	3.6 ± 1.4	3.6 ± 1.4	0.892	3.7 ± 1.5	4.1 ± 1.3	0.114
CDAI	16.7 ± 15.1	17.5 ± 13.5	0.707	18.6 ± 17.2	20.2 ± 12.6	0.609
SDAI	18.4 ± 15.9	18.2 ± 14.2	0.943	20.4 ± 18.0	22.1 ± 13.6	0.616
HAQ-DI	0.7 ± 0.8	0.7 ± 0.8	0.594	0.7 ± 0.7	0.8 ± 0.7	0.236

Data are presented as mean±S.D. or percentage, unless otherwise specified.

ACPA: anti-citrullinated protein antibody; b/tsDMARD: bio/targeted synthetic disease-modifying anti-rheumatic drug; CDAI: Clinical Disease Activity Index; CRP: C-reactive protein; DAS28-CRP: Disease Activity Score 28 with CRP; EGA: Evaluator Global Assessment; GC: glucocorticoid; HAQ-DI: Health Assessment Questionnaire Disability Index; IQR: interquartile range (Q1–Q3); MMP-3: matrix metalloproteinase-3; MTX: methotrexate; PGA: Patient Global Assessment; RF: rheumatoid factor; SDAI: Simplified Disease Activity Index; SJC: swollen joint count; TJC: tender joint count; TNF: tumor necrosis factor.

Among the matched patients, 10 discontinued baricitinib within the first 24 weeks for the following reasons: inefficacy (three patients), infections (three patients), malignancy (two patients), drug-induced interstitial lung disease (one patient) and death (one patient). Additionally, six patients discontinued treatment between 24 and 52 weeks for the following reasons: inefficacy (two patients), diverticulitis of the colon (one patient), lymphoproliferative disorder (one patient), cerebral haemorrhage (one patient) and malignancy (one patient). Furthermore, six patients discontinued sarilumab within the first 24 weeks for the following reasons: inefficacy (three patients), nausea (one patient), urticaria (one patient) and self-discontinuation (one patient). Additionally, eight patients discontinued treatment between 24 and 52 weeks for the following reasons: inefficacy (five patients), injection site reaction (one patient), self-discontinuation (one patient) and pregnancy (one patient).

**Figure 1.**
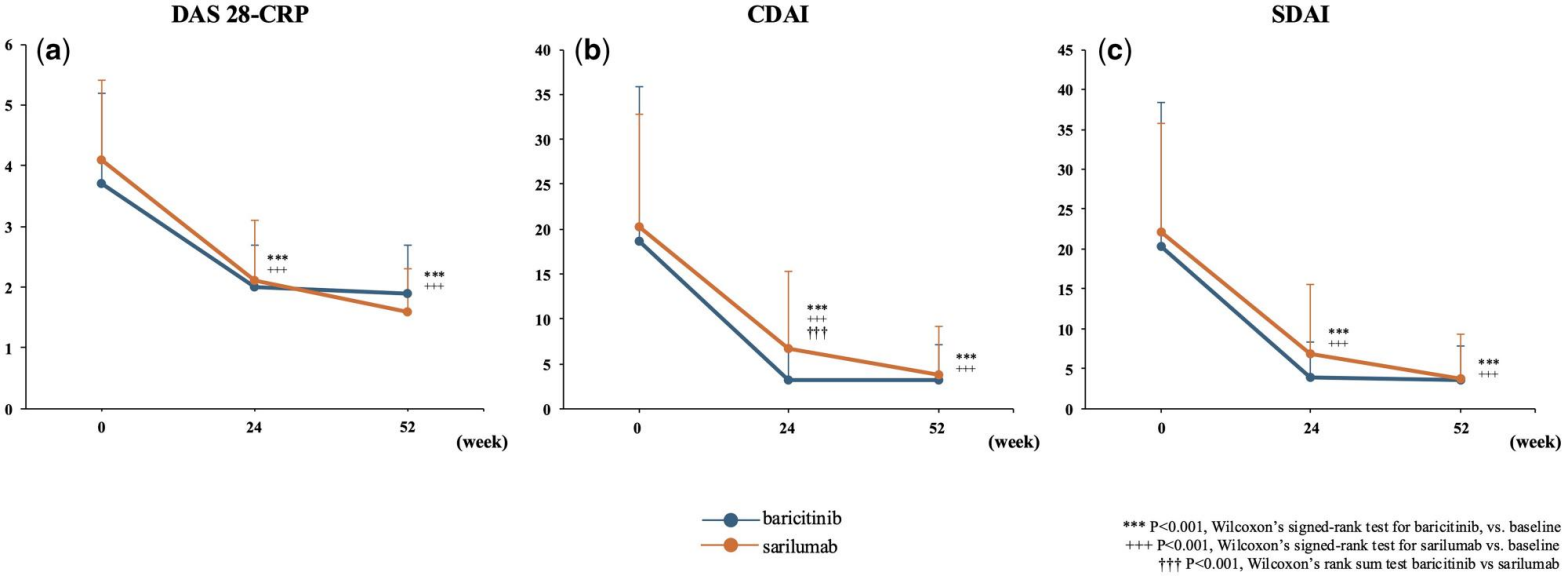
Comparison of disease activity. (**a**) Mean DAS28-CRP. (**b**) Mean CDAI. (**c**) Mean SDAI. Error bars represent the S.D. The number of patients with matched data for each time point was as follows: baseline (*n* = 46 for each group), 24 weeks (*n* = 36 for baricitinib, *n* = 40 for sarilumab) and 52 weeks (*n* = 30 for baricitinib, *n* = 32 for sarilumab). DAS28-CRP: Disease Activity Score 28 with CRP; CDAI: Clinical Disease Activity Index; SDAI: Simplified Disease Activity Index

In the baricitinib group, the average DAS28-CRP significantly decreased from baseline to 24 weeks (from 3.7 ± 1.5 to 2.0 ± 0.7; *P* < 0.001; Fig. 1a). This significant reduction was maintained throughout 52 weeks, with the scores remaining low at 1.89 ± 0.78 (*P* < 0.001; Fig. 1a). Similarly, in the sarilumab group, the average DAS28-CRP significantly decreased from baseline to 24 weeks (from 4.1 ± 1.3 to 2.1 ± 1.0; *P* < 0.001; Fig. 1a). This reduction was also sustained up to 52 weeks, with the scores continuing to be low at 1.6 ± 0.7 (*P* < 0.001; Fig. 1a). However, no significant differences in treatment effectiveness were observed between the two groups at 24 and 52 weeks.

In the baricitinib group, significant reductions were observed not only in DAS28-CRP but also in the CDAI and the SDAI from baseline to 24 weeks (CDAI: from 18.6 ± 17.2 to 3.2 ± 3.5, *P* < 0.001; Fig. 1b; SDAI: from 20.4 ± 18.0 to 3.9 ± 4.4, *P* < 0.001; Fig. 1c). These significant improvements were maintained throughout 52 weeks, with CDAI and SDAI remaining low (3.2 ± 4.0 and 3.6 ± 4.2, respectively, *P* < 0.001; Fig. 1b and c). Similarly, in the sarilumab group, reductions in the CDAI and SDAI from baseline to 24 weeks were significant (CDAI: from 20.2 ± 12.6 to 6.7 ± 8.6, *P* < 0.001; Fig. 1b; SDAI: from 22.2 ± 13.6 to 6.9 ± 8.7, *P* < 0.001; Fig. 1c). These measures of disease activity also continued to be significantly lower at 52 weeks (3.7 ± 5.5 and 3.8 ± 5.5, respectively, *P* < 0.001; Fig. 1b and c), indicating the sustained effectiveness of the treatment. At 24 weeks, the CDAI in the baricitinib group was significantly lower than that in the sarilumab group; however, this significant difference in CDAI and any trend in SDAI were not observed at 52 weeks.

**Figure 2.**
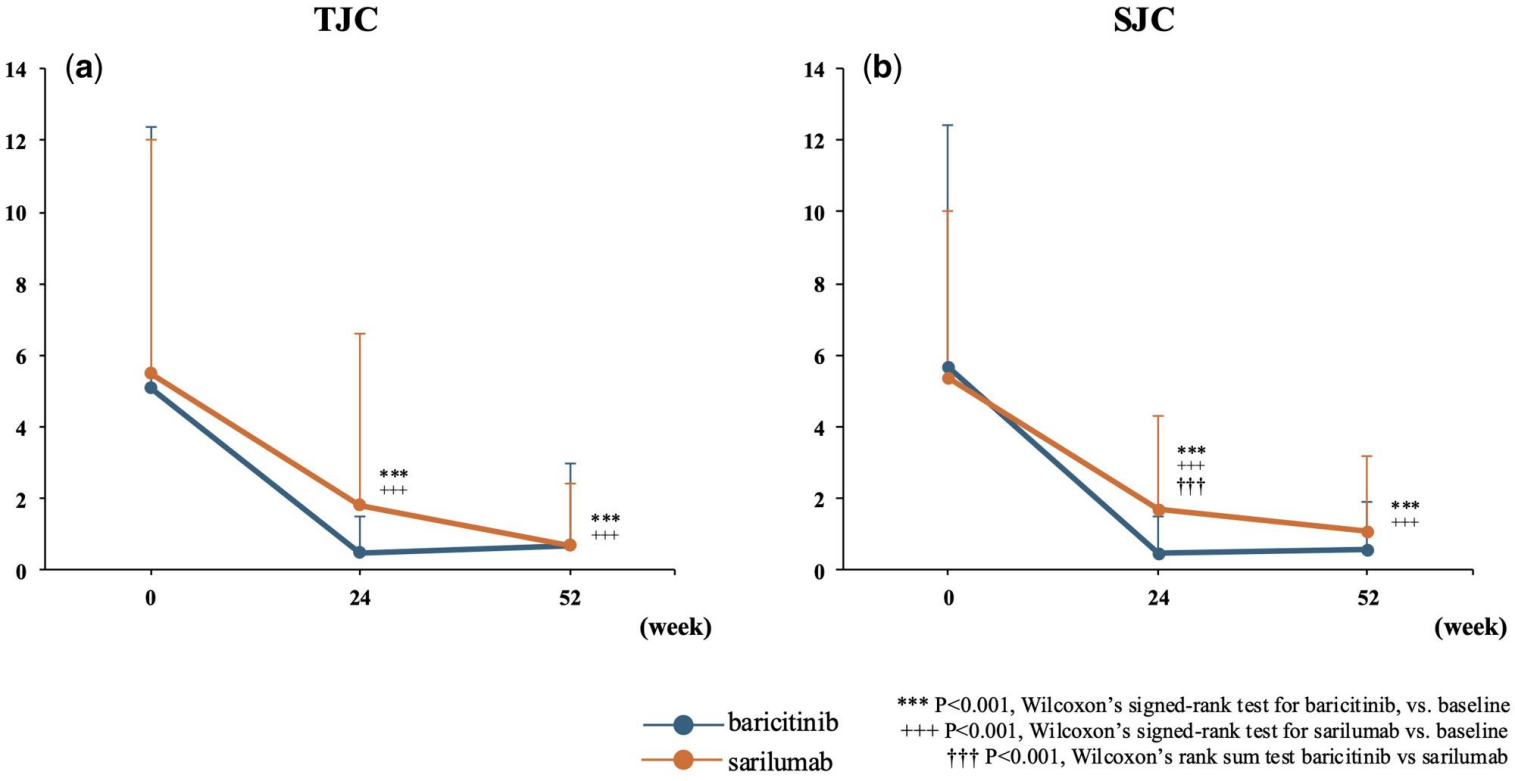
Comparison of TJC and SJC. (**a**) Mean TJC. (**b**) Mean SJC. Error bars represent the S.D. The number of patients with matched data for each time point was as follows: baseline (*n* = 46 for each group), 24 weeks (*n* = 36 for baricitinib, *n* = 40 for sarilumab) and 52 weeks (*n* = 30 for baricitinib, *n* = 32 for sarilumab). TJC: tender joint count; SJC: swollen joint count

**Figure 3.**
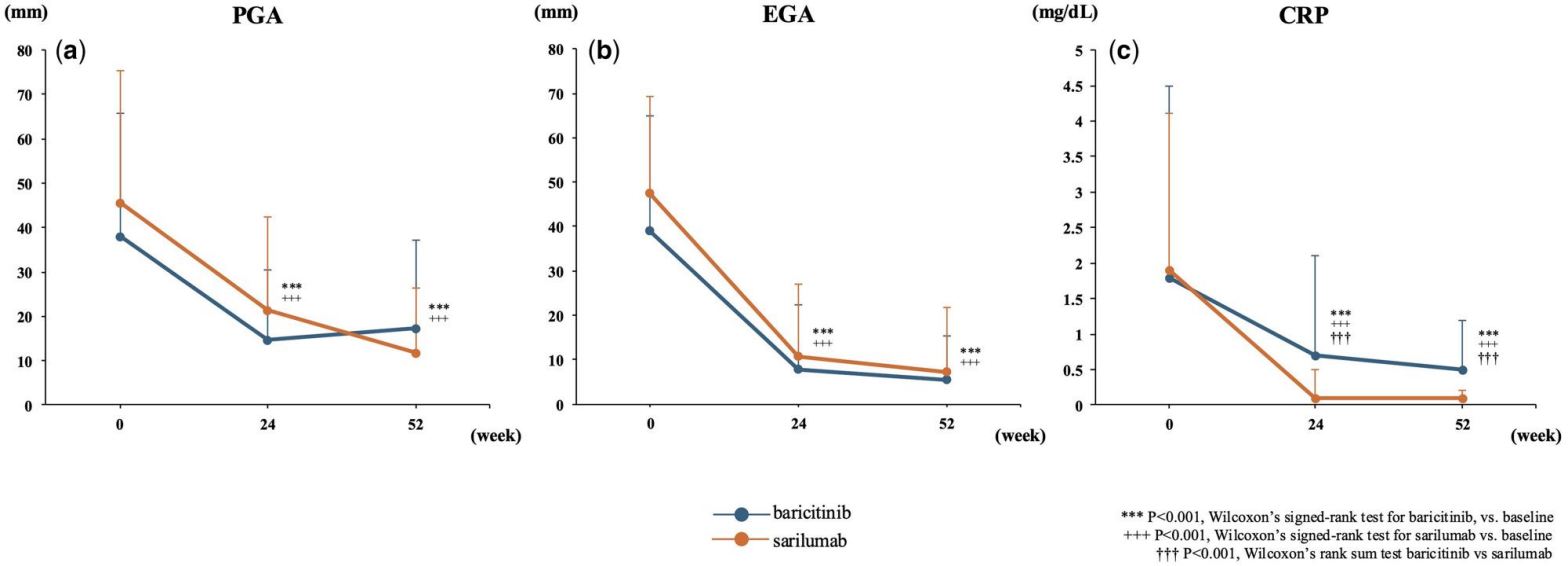
Comparison of PGA, EGA and CRP. (**a**) Mean PGA. (**b**) Mean EGA. (**c**) Mean CRP. Error bars represent the S.D. The number of patients with matched data for each time point was as follows: baseline (*n* = 46 for each group), 24 weeks (*n* = 36 for baricitinib, *n* = 40 for sarilumab) and 52 weeks (*n* = 30 for baricitinib, *n* = 32 for sarilumab). PGA: Patient Global Assessment; EGA: Evaluator Global Assessment of Disease Activity

Over the course of 52 weeks, analyses of DAS28-CRP components, including TJC, SJC, PGA, EGA and CRP levels, were undertaken in the baricitinib and sarilumab groups. At 24 weeks, the baricitinib group saw a reduction in TJCs, from 5.1 ± 7.3 to 0.5 ± 1.0 (*P* < 0.001; Fig. 2a); SJCs, from 5.7 ± 6.7 to 0.5 ± 1.0 (*P* < 0.001; Fig. 2b); PGA, from 38.0 ± 27.6 to 14.8 ± 15.7 (*P* < 0.001; Fig. 3a); EGA, from 39.1 ± 25.9 to 7.8 ± 14.4 (*P* < 0.001; Fig. 3b) and CRP levels, from 1.8 ± 2.7 to 0.7 ± 1.4 (*P* < 0.05; Fig. 3c). Similarly, the sarilumab group experienced changes in TJCs, from 5.5 ± 6.5 to 1.8 ± 4.8 (*P* < 0.01; Fig. 2a); SJCs, from 5.4 ± 4.6 to 1.7 ± 2.6 (*P* < 0.001; Fig. 2b); PGA, from 45.6 ± 29.7 to 21.3 ± 21.0 (*P* < 0.001; Fig. 3a); EGA, from 47.4 ± 22.0 to 10.7 ± 16.2 (*P* < 0.001; Fig. 3b); and CRP levels, from 1.9 ± 2.2 to 0.1 ± 0.4 (*P* < 0.001; Fig. 3c). At 24 weeks, baricitinib showed a significantly better improvement in SJCs than sarilumab. Extending the analysis to 52 weeks, the baricitinib group maintained or improved their reductions, with TJC at 0.7 ± 2.3 (*P* < 0.001; Fig. 2a), SJC at 0.6 ± 1.3 (*P* < 0.001; Fig. 2b), PGA at 17.3 ± 20.0 (*P* < 0.001; Fig. 3a), EGA at 5.3 ± 10.0 (*P* < 0.001; Fig. 3b) and CRP levels at 0.5 ± 0.7 (*P* < 0.01; Fig. 3c). The sarilumab group showed comparable results, with TJC at 0.7 ± 1.7 (*P* < 0.001; Fig. 2a), SJC at 1.1 ± 2.1 (*P* < 0.001; Fig. 2b), PGA at 11.9 ± 14.4 (*P* < 0.001; Fig. 3a), EGA at 7.2 ± 14.5 (*P* < 0.001; Fig. 3b) and CRP at 0.1 ± 0.1 (*P* < 0.001; Fig. 3c). No significant differences in these components, except for CRP, were observed between the two groups. Additionally, an analysis of changes in disease activity scores from baseline showed no statistically significant differences between the baricitinib and sarilumab groups at 24 weeks and 52 weeks. These changes are further illustrated in Supplementary Fig. S1, available at *Rheumatology Advances in Practice* online.

**Figure 4.**
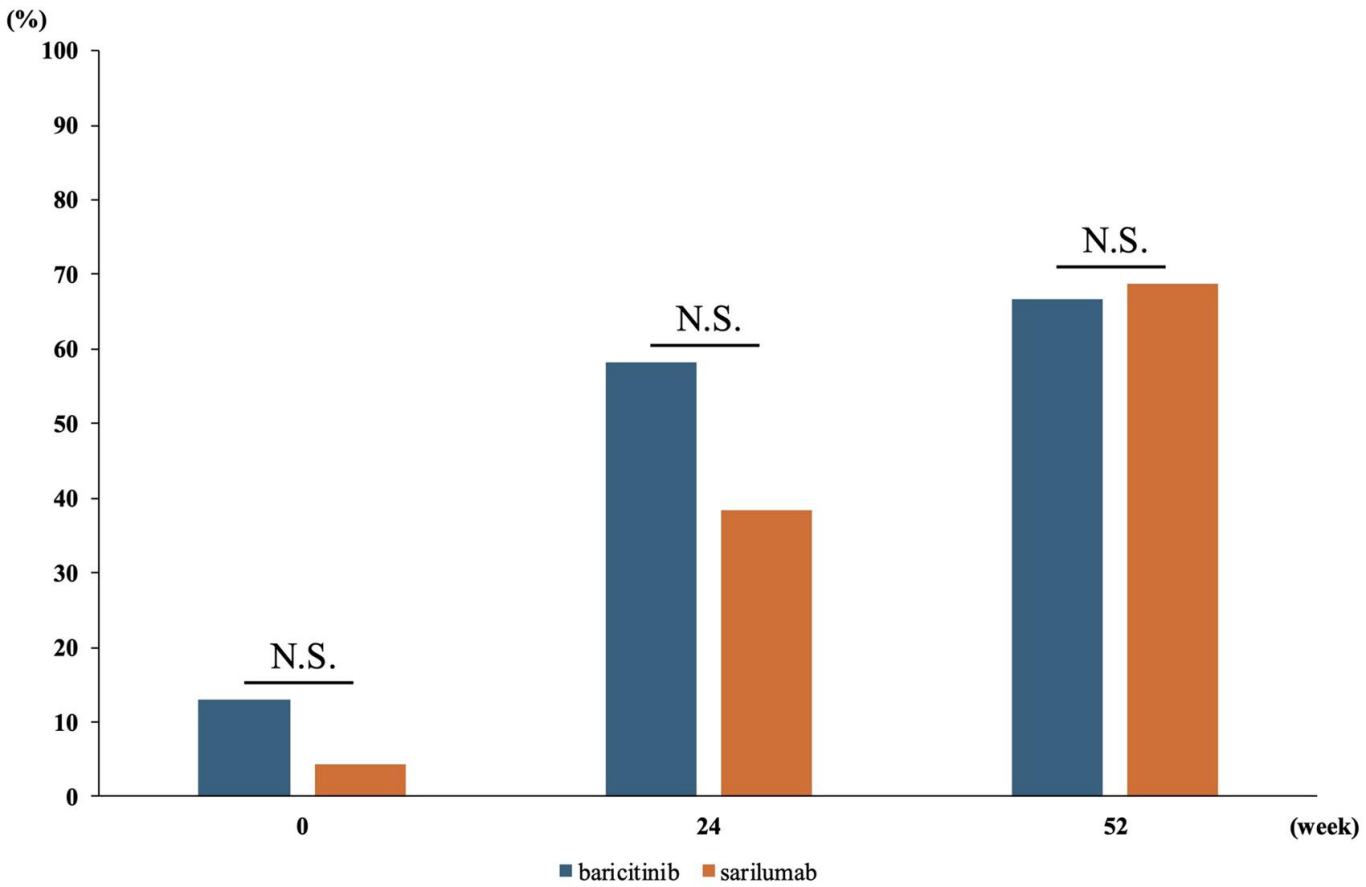
Boolean 2.0 remission rate. The number of patients with matched data for each time point was as follows: baseline (*n* = 46 for each group), 24 weeks (*n* = 36 for baricitinib, *n* = 40 for sarilumab) and 52 weeks (*n* = 30 for baricitinib, *n* = 32 for sarilumab). N.S.: not significant

In terms of Boolean remission 2.0, the proportion of patients who achieved remission in the baricitinib and sarilumab groups were 6/46 (13%) and 2/46 (4%) at the start of treatment, 21/36 (58%) and 15/39 (38%) at 24 weeks, and 20/30 (67%) and 22/32 (69%) at 52 weeks, respectively (Fig. 4). No significant differences were observed between the two groups.

**Figure 5.**
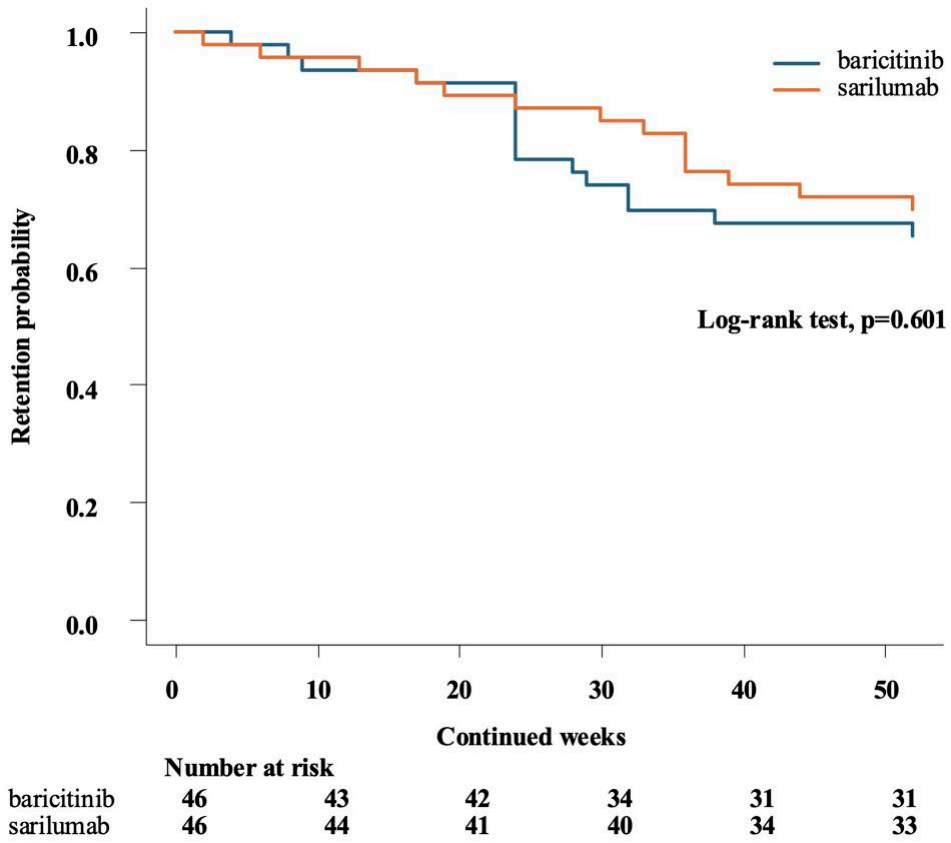
Kaplan–Meier survival curve comparing drug retention rates between treatment groups over 52 weeks. Kaplan–Meier survival curves showing drug retention over a 52-week period for two treatment groups: baricitinib and sarilumab. The *x*-axis represents time in weeks, and the *y*-axis represents the cumulative probability of drug retention. Numbers below the *x*-axis indicate the number of patients at risk at each time point for each treatment group

The Kaplan–Meier curves of drug retention for the baricitinib and sarilumab groups over 52 weeks are shown in Fig. 5. There was no significant difference in drug retention rates between the two groups (log-rank test, *P* = 0.601).

## Discussion

In this study, using real-world data and propensity score matching to compare baricitinib and sarilumab, we observed significant reductions in the CDAI with baricitinib at 24 weeks. However, at 24 and 52 weeks, no significant differences were observed in the measures of disease activity, except for CDAI at 24 weeks.

Previous reports have compared baricitinib and tocilizumab using propensity score matching for direct efficacy comparison, similar to the present study; while the study did not specifically address differences in the CDAI at 24 weeks, it reported a superior improvement rate in PGA for baricitinib [8]. They suggested that this difference might stem from baricitinib’s broader inhibition of JAK-dependent cytokines beyond IL-6, such as granulocyte-macrophage colony-stimulating factor, which could contribute to additional pain relief and improvements in patient-reported outcomes. This broader cytokine inhibition could explain why baricitinib achieved a higher rate of Boolean remission in their study, despite similar levels of control over joint inflammation and CRP. However, no significant differences were observed in PGA between the two treatments in our study. This may be due to our study population’s relatively low baseline PGA, which left less room for improvement. In addition, the PGA is influenced by various factors, such as age, pain, fatigue, stiffness and HAQ [12]. In this study, the patients were elderly, and it is likely that improvements in SJC did not significantly lower the PGA, possibly due to other factors like age-related conditions or persistent symptoms such as pain and fatigue.

Further analysis at the disease activity component level showed that baricitinib significantly reduced SJC at 24 weeks, while sarilumab achieved significant reductions in CRP at both 24 and 52 weeks. These results suggest that baricitinib’s effects may extend beyond systemic inflammation, directly targeting joint symptoms associated with disease activity, as reflected in the reduction of SJC. Since SJC is a direct measure of joint inflammation, this reduction may indicate greater clinical effectiveness in managing the localized joint symptoms of RA, potentially leading to improvement in patient quality of life and overall physical function.

While both baricitinib and sarilumab have mechanisms that can lead to reductions in CRP due to their effects on IL-6 signalling pathways, the clinical implications may differ. Baricitinib’s broader inhibition of cytokines beyond IL-6, due to its action on JAK1 and JAK2, may contribute to its significant reduction in SJC at 24 weeks, which is directly associated with clinical improvement. In contrast, sarilumab’s more targeted IL-6 inhibition leads to a pronounced reduction in CRP, a systemic inflammation marker; however, this effect may not directly translate to relief of clinical symptoms as effectively as joint-specific improvements. Thus, while both drugs show efficacy, their clinical effectiveness may differ depending on the observed outcomes.

In contrast, sarilumab’s targeted IL-6 inhibition primarily reduces systemic inflammation, as evidenced by decreased CRP levels. Although CRP is a valuable marker for tracking systemic inflammation, its reduction alone may not directly translate to improvements in joint symptoms as effectively as SJC reduction does. Therefore, while both drugs are effective in managing RA, the observed differences in SJC and CRP reductions might imply that baricitinib offers additional clinical benefits for patients with significant joint involvement. In contrast, sarilumab’s impact is more focused on systemic inflammatory response.

The high discontinuation rates in our study may be attributed to the older age and complex comorbidities of our study population. Older patients with longer disease duration and higher disease severity are at greater risk of adverse events or insufficient treatment response, leading to higher dropout rates. This selective dropout may introduce bias by excluding patients with poorer responses or greater susceptibility to adverse effects, potentially resulting in overestimating treatment efficacy in the remaining cohort. We used the LOCF method to address this, acknowledging its limitations, especially with non-random dropout patterns. Additionally, previous studies have reported similar discontinuation patterns among older patients treated with baricitinib [13], consistent with the findings of our study.

Reports suggest that in patients with late-onset RA, an increase in the concentrations of various inflammatory cytokines, excluding IL-6, is observed [14]. This implies that for elderly patients with RA, broader cytokine inhibition, rather than solely blocking IL-6, may be a more appropriate therapeutic strategy. The disappearance of significant differences in disease activity at 52 weeks suggests that the long-term continuation of treatment is an important aspect of managing RA. This finding underscores the value of sustained treatment regimens for achieving and maintaining control of disease activity, highlighting the importance of long-term management strategies in RA care.

However, it is also important to weigh the potential risks of JAK inhibitors, especially in elderly patients who may be more susceptible to adverse events like major cardiovascular events and malignancies. The drug retention rates over 52 weeks showed no significant differences between the two groups. While the reasons for discontinuation were comparable between the groups, three cases of malignancy were observed in the baricitinib group. Maintaining long-term efficacy while ensuring safety is critically important in the current treatment strategies for RA.

This study has several limitations that should be considered when interpreting the results. First, due to its retrospective design and the fixed sample size, an *a priori* power calculation was not feasible. Instead, we conducted a *post hoc* power analysis, which resulted in a power of 0.72. Although this falls short of the conventional threshold of 0.8, we believe it provides a reasonable basis for interpreting the findings within real-world clinical constraints. Additionally, performing PSM reduced the sample size to 46 cases in each group, which may have further limited statistical power. However, we believe PSM allowed for a more balanced comparison between patient groups and yielded more reliable group comparisons. Second, although baricitinib showed greater clinical effectiveness in disease activity measures at early time points, we found no statistically significant difference in the changes in disease activity scores from baseline between the two groups. This suggests that conclusions regarding baricitinib’s early superiority over sarilumab should be approached cautiously. Further studies with larger sample sizes are needed to robustly evaluate these findings. Lastly, this study did not include imaging assessments, which could provide insights into structural changes. Previous reports suggest that disease activity measures do not always correlate with structural changes observed in radiographic imaging [15]. Therefore, it is possible that differences in long-term effects on joint structures between the two treatments were not captured in this study despite comparable efficacy observed in disease activity scores. Since structural changes may become more apparent over extended periods, further longitudinal studies including imaging assessments are warranted to clarify potential long-term differences between these treatments.

In conclusion, this study expands upon existing knowledge by comparing JAK and IL-6 inhibitors, further enhancing our understanding of their unique properties and contributions to improved clinical outcomes. We found that sarilumab caused a greater reduction in CRP levels at all observed time points; however, at 24 weeks, baricitinib showed a significant improvement in the CDAI, which does not incorporate CRP as a component. By 52 weeks, this difference had disappeared, indicating the efficacy of both medications. This suggests that continued treatment with either drug can sustain the suppression of RA disease activity.

## Supplementary Material

rkaf006_Supplementary_Data

## Data Availability

The data underlying this article are available upon request and are subject to a confidentiality agreement due to the sensitive nature of the data.
