# Supplementary figures and images for: Effectiveness of baricitinib versus sarilumab on disease activity in patients with RA: a propensity score matching study
Source: Rheumatol Adv Pract. 2025 Jan 10;9(1):rkaf006. doi: 10.1093/rap/rkaf006 (PMC11829163; doi:10.1093/rap/rkaf006)

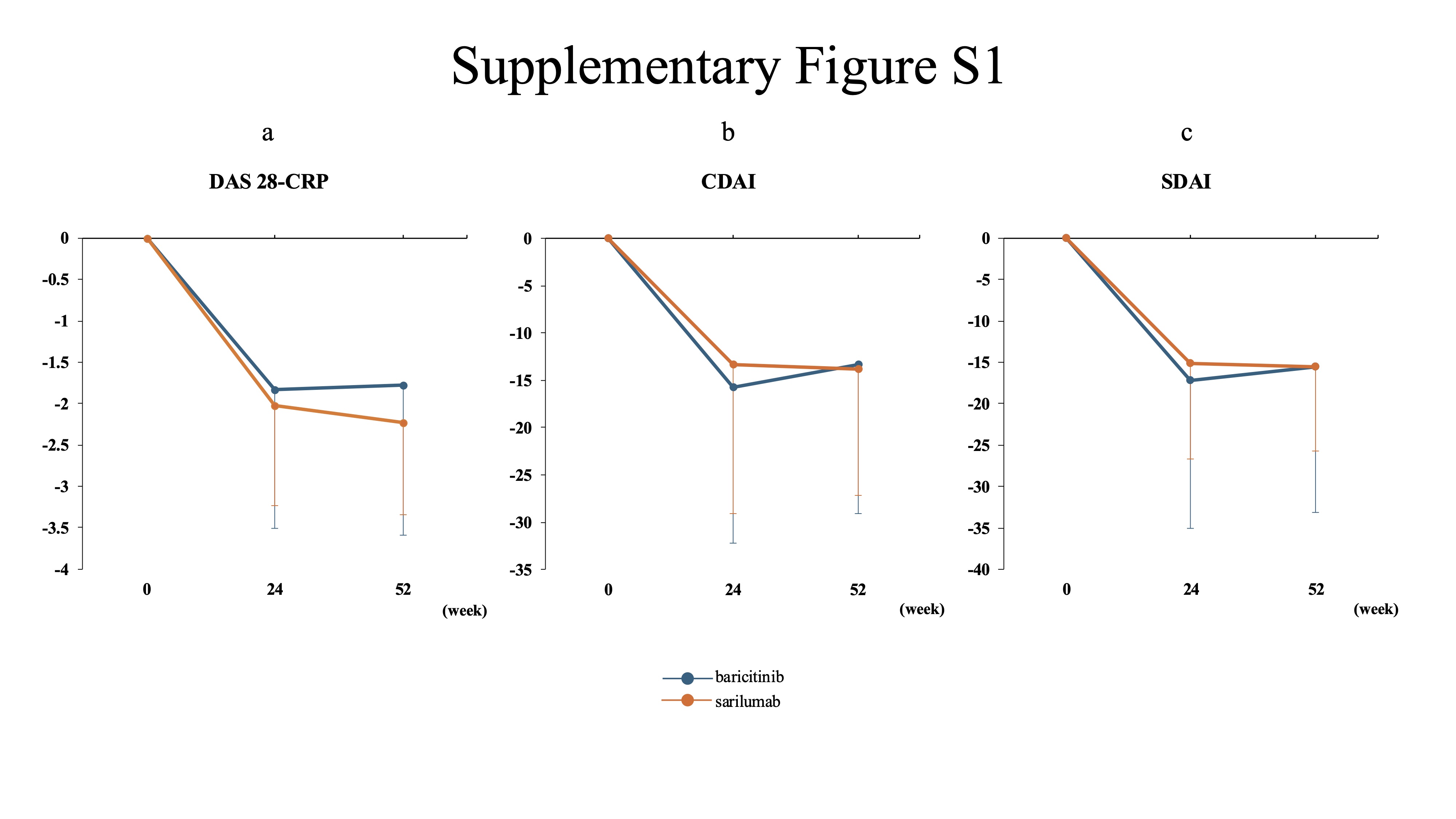

Supplement: rkaf006_Supplementary_Data [file rkaf006_supplementary_data.jpeg]
